# Effectiveness of integrated care model for type 2 diabetes: A population-based study in Reggio Emilia (Italy)

**DOI:** 10.1371/journal.pone.0194784

**Published:** 2018-03-27

**Authors:** Paola Ballotari, Francesco Venturelli, Valeria Manicardi, Francesca Ferrari, Massimo Vicentini, Marina Greci, Fabio Pignatti, Simone Storani, Paolo Giorgi Rossi

**Affiliations:** 1 Epidemiology Unit, Local Health Authority of Reggio Emilia-IRCCS, Reggio Emilia, Italy; 2 Specialization school of Hygiene and Preventive Medicine, Department of Biomedical, Metabolic and Neural Sciences, University of Modena and Reggio Emilia, Modena, Italy; 3 Clinical and Experimental Medicine PhD Program, University of Modena and Reggio Emilia, Modena, Italy; 4 Department of Internal Medicine, Hospital of Montecchio, Local Health Authority of Reggio Emilia-IRCCS Reggio Emilia, Italy; 5 Primary Health Care, Local Health Authority of Reggio Emilia-IRCCS Reggio Emilia, Italy; Shanghai Diabetes Institute, CHINA

## Abstract

**Aims:**

To compare the effectiveness of integrated care with that of the diabetes clinic care model in terms of mortality and hospitalisation of type 2 diabetes patients with low risk of complications.

**Methods:**

Out of 27234 people with type 2 diabetes residing in the province of Reggio Emilia on 31/12/2011, 3071 were included in this cohort study as eligible for integrated care (i.e., low risk of complications) and cared for with the same care model for at least two years. These patients were followed up from 2012 to 2016, for all-cause and diabetes-related mortality and hospital admissions. We performed a Poisson regression model, using the proportion of eligible patients included in the integrated care model for each general practitioner as an instrumental variable.

**Results:**

1700 patients were cared for by integrated care and 1371 by diabetes clinics. Mortality rate ratios were 0.83 (95%CI 0.60–1.13) and 0.95 (95%CI 0.54–1.70) for all-cause and cardiovascular mortality, respectively, and incidence rate ratios were 0.90 (95%CI 0.76–1.06) and 0.91 (95%CI 0.69–1.20) for all-cause and cardiovascular disease hospitalisation, respectively.

**Conclusion:**

For low risk patients with type 2 diabetes, the integrated care model involving both general practitioner and diabetes clinic professionals showed similar mortality and hospitalisation as a model with higher use of specialized care in an exclusively diabetes clinic setting.

## Introduction

The prevalence of type 2 diabetes (T2D) is increasing worldwide [1]. In Italy, it has reached 6% of the adult population [2,3]. T2D increases the risk of many severe diseases and of death [4]. Control of glycaemia and early identification of possible complications of diabetes are effective ways of reducing the burden of disease [5]. In Italy, normal treatment of T2D requires a referral by a general practitioner (GP) to a diabetes clinic (DC) for diagnostic confirmation, therapy, prevention and early diagnosis of complications through close patient follow up by a team of diabetologists, nurses and dieticians, and for scheduling of regular check-ups. This model of care is quite intensive and resource-consuming, and many diabetes clinics cannot take care of all the patients in their areas. Since 2006, a new model of care called integrated care (IC) was introduced, a shared care model involving both GPs and DC professionals [6]. The IC model targets low risk T2D patients, i.e., those with good glycaemic control who do not use rapid-acting insulin and are not suffering from mild or severe complications. The care plan envisages visit every two years at the DC, as well as quarterly check-ups performed by a GP. Participation in IC is voluntary for both patients and physicians. The IC model is consistent with the WHO recommendations regarding the management of chronic diseases [7].

The effectiveness of a shared care health service has been debated in a recent review, which found that shared care probably has limited or no effect on physical clinical outcomes, except for a tendency towards improved blood pressure, and little or no difference in hospital admission rates [8]. The review included studies focusing on diabetes [9–17].

Although randomized trials are the gold standard for comparing treatments or interventions, comparative effectiveness research has emphasised the use of observational studies to investigate treatment or intervention choices within larger and more representative populations [18]. However, strong biases can affect the results of observational studies where the allocation to one treatment or another is based on the physician’s assessment of prognostic factors [19,20].

In the comparison between the DC and IC models for T2D, two limitations can lead to biases in the results. First, the eligibility criteria for the new model of care (i.e., IC) define a very low risk group of patients compared to those in the DC model. This selection bias could be partially overcome by limiting the comparison only to those T2D patients who meet the IC criteria. The second bias regards confounding by indication bias (or treatment selection bias); physician and patient preferences and opportunities as well as patient frailty, lack of motivation or low self-efficacy, and/ or social or economic deprivation could be related to both treatment selection and outcome. As a result, residual confounding is expected when traditional methods of adjustment (regression or propensity score analysis) are used to compare the treatment effects [21]. One way to overcome this is to use the instrumental variable approach [22]. Similar to randomisation, an instrumental (or endogenous) variable is related to the treatment selection but is not directly related to the outcome. Its occurrence creates a natural experiment, i.e., a situation that allows for random or seemingly random assignment and can overcome the effect of unmeasured confounders.

In the province of Reggio Emilia (northern Italy), the IC model has been implemented gradually; while GP participation in some districts has increased rapidly, very few GPs in other districts initially agreed to participate. Furthermore, during the study period, each GP had a different level of engagement with IC. Therefore, the percentage of eligible patients who were cared for by the IC model varied from 0 to 100%, depending on the GP. Thus, the gradual implementation over time and across GPs has created a natural experiment that can be used to examine the comparative effectiveness of alternative care models. Moreover, the Reggio Emilia diabetes register (REDR) [2] permits conducting observational studies on a cohort of prevalent T2D patients to investigate differences in outcome measures.

This study aims to compare the effectiveness of IC with that of the DC care model in terms of mortality and hospitalisation rates of T2D patients with low risk of complications over a five-year follow-up period. The hypothesis is that IC is not inferior to the DC care model in controlling diabetes-related morbidity, while it should lead to lower resource consumption and be less time consuming for patients.

## Materials and methods

### Study population and study design

The province of Reggio Emilia is situated in northern Italy and has approximatively 530,000 inhabitants, 300 general practitioners, 6 outpatient diabetes clinics and 1 diabetes inpatient unit in the main hospital.

This cohort study included people residing in the province of Reggio Emilia on December 31, 2011, diagnosed with T2D, under the age of 85 and cared for by the same care model for at least two years. Accordingly, three groups were created: 1) cared for by diabetes outpatient clinics (DC); 2) integrated care (IC); 3) Other-group (neither DC nor IC, maybe only cared for by own GP but also voluntary opt-outs who turn to private care or neglected patients).

Patients were followed up from 1 January 2012 to 31 December 2016, for all-cause and diabetes-related mortality and hospital admissions.

T2D status was retrieved from the Reggio Emilia diabetes register (REDR).

REDR is a validated register created by the deterministic linkage of six routinely collected data sources through a definite algorithm able to ascertain cases and to distinguish the type of diabetes and model of care [2]. The date of inclusion in the register is the date when a person first meets one of the following inclusion criteria: (1) Disease-specific exemption database: exemption from co-payments due to diabetes; (2) Hospital discharge database: hospitalisation with diabetes diagnosis in whichever position by ICD-9 (International Classification of Diseases Clinical Modification, 9th Edition) codes 250.xx, 357.2x, 362.0x, 366.41, or 648.0x, excluding MDC14; (3) Biochemistry laboratory database: two glycated haemoglobin (HbA1c) tests > = 6.5% (48 mmol/mol) or one HbA1c> = 6.5% and (48 mmol/mol) test followed by a fasting blood sugar test> = 126 mg/dl; (4) Drug prescription databases: either at least two redeemed prescriptions for anti-diabetic drugs in a pharmacy or one prescription directly distributed by the hospital; (5) Diabetes outpatient clinics database: diagnosis by a diabetologist; (6) Mortality registry: cause of death by ICD-10 (International Classification of Diseases, 10th Edition) codes E10 –E14. Women with gestational diabetes or women receiving treatment for polycystic ovarian syndrome or obesity are excluded from the register. Cases initially notified to the registry through record linkage are retained if they are clinically confirmed by a diabetologist or another physician.

Exposure definition was assessed in the 2009–2011 period (see S1 Fig for study timeline chart). Patients were retained in the study and assigned to IC or DC care if they remained in the same care model for at least two years before the start of follow up. Shifts from one care model to the other after the start of follow up did not modify the exposure allocation, because the worsening of the disease would imply shifting from IC to DC. Thus, most of the investigated outcomes tended to occur in DC even if the worsening began during IC.

T2D patients were classified as eligible for IC according to regional guidelines criteria [6]. The criteria were assessed in the period before the start of follow up: 1) no hospital admissions for diabetes-related complications in the previous two years, i.e., from 2009 to 2010 (see S1 Table for list of first diagnosis codes considered); 2) no mild or severe renal complications (i.e, those with the last glomerular filtrate before start of follow up> = 60 ml/min/1.73m^2^, considering 2010–2011 measurements); 3) HbA1c on target (last value before start of follow up < = 7% (53 mmol/mol) if aged less than 75 or < = 8% (64 mmol/mol) if aged equal to or more than 75, considering 2010–2011 measurements); 4) no use of rapid-acting insulin in 2011. Other covariates were assessed in 2011.

### Outcomes and covariates

Data on deaths and cause of death were collected from the Reggio Emilia mortality registry. Causes were classified according to the International Classification of Diseases, 10th Revision, and detailed as all-causes (A00-Z99), cardiovascular disease (I00-I99), acute myocardial infarction (I21-I22, I24.8-I24.9), cerebrovascular diseases (I60-I69), diabetes (E10-E14) or renal diseases (N00-N39). Data on hospital admission, from the Hospital discharge database, were classified according to the International Classification of Diseases, 9th Revision, and detailed as all-causes (001-V89), cardiovascular diseases (390–459), acute myocardial infarction or AMI (410), cerebrovascular diseases (430–438), hypoglycaemic coma (250.3), acute hyperglycaemic complications (250.1–250.2), non-traumatic lower limb amputations (drg 113, 114 and 285), renal complications (250.4, 581.81, 584, 585, 586, 791.0), or ocular complications (250.5, 361.0, 361.9, 362.0, 362.1, 362.83, 364.42, 365.44, 365.6, 366.1, 366.41, 369, 377, 379.2). Day hospital admissions were excluded and only the main diagnosis was considered.

The covariates were sex, citizenship status, age, district of residence, time since diagnosis, HbA1c, body mass index (BMI), triglycerides, low density lipoprotein cholesterol (LDL), glomerular filtration rate (GFR), and glucose lowering medication. When not available, we estimated GFR by using serum creatinine according to Modification of Diet in Renal Disease (MDRD) Study equation [23].

### Statistical analysis

We compared patients’ baseline characteristics stratified by current care model using the χ2 or Student t test, as appropriate. After classifying GPs according to the proportion of their patients eligible for IC model and those who were actually included in IC, we attributed this variable to each eligible patient, and we divided eligible patient population into quartiles. Eligible patients’ baseline characteristics are reported according to quartile distribution to show the comparability of the population included in the 1^st^ quartile (i.e., assigned to GPs with lower adoption of IC) and the 4^th^ quartile (i.e., assigned to GPs with higher adoption of IC).

To measure the current differences in the care models, we calculated crude and standardised rates (by sex and age) and 95% confidence intervals (95%CI) for mortality and hospital admission outcomes, the latter considering all discharges occurring for the same patient in the follow-up period.

The conceptual non-inferiority hypothesis of IC compared to the DC care model was assessed by calculating and comparing RRs and related 95%CI through multivariate models in order to reduce the effect of a potential bias. First, we used a Poisson regression model, calculating mortality and hospitalisation rate ratios (RRs) and 95%CI only among IC-eligible patients cared for by DC or IC models. Persons-time was calculated as the difference between the start of the follow-up period (January 1, 2012) and the date of the event (death or first hospitalisation), moving out of the province or end of follow up (December 31, 2016), whichever came first. The DC care model was used as a reference and the covariates were sex, citizenship, age, years since diagnosis, the last two as continuous variables. Clinical conditions at baseline (HbA1c, triglycerides, BMI, GFR) were not included in the multivariate analysis because the values were balanced between groups. The only possible confounder was LDL, but the high proportion of missing values made it impossible to use to adjust the models. In this analysis we excluded the Other-group (only the number of events was reported) because it was not a care model foreseen by the guideline, but a residual group, the consequence of the incomplete application of either of the other two care models. Relative risks were not calculated for diabetes-related causes for which the number of expected events in at least one of the two care models was less than five.

Secondly, we performed a Poisson regression model, using our instrumental variables (i.e., the proportion of GP-eligible patients actually included in the IC model) as a main predictor. This variable was attributed individually to all eligible patients, and the model took into account that observations within the same GP’s patient list were not independent (STATA command Poisson, cluster option). The other covariates were the same as the above multivariate model.

The analyses were performed using the STATA statistical package Version 13.0

### Ethical approval

This is an observational study and the data were collected retrospectively. The Local Health Authority of Reggio Emilia was responsible for collecting and processing the data. The Reggio Emilia Diabetes Registry was approved by the provincial Ethics Committee on July 23, 2014 (Comitato Etico Provinciale of Reggio Emilia, now Comitato Etico AVEN, after being merged with the other Ethics Committees of the Modena, Piacenza and Parma provinces http://www.aou.mo.it/ComitatoEticoAVEN). The aim of the study is consistent with the specific objective of the REDR as approved by the Ethics Committee. In accordance with the Italian privacy law, no patient or parental consent is required for large retrospective population-based studies approved by the competent Ethics Committee if data are published only in aggregated form.

## Results

**Fig 1:**
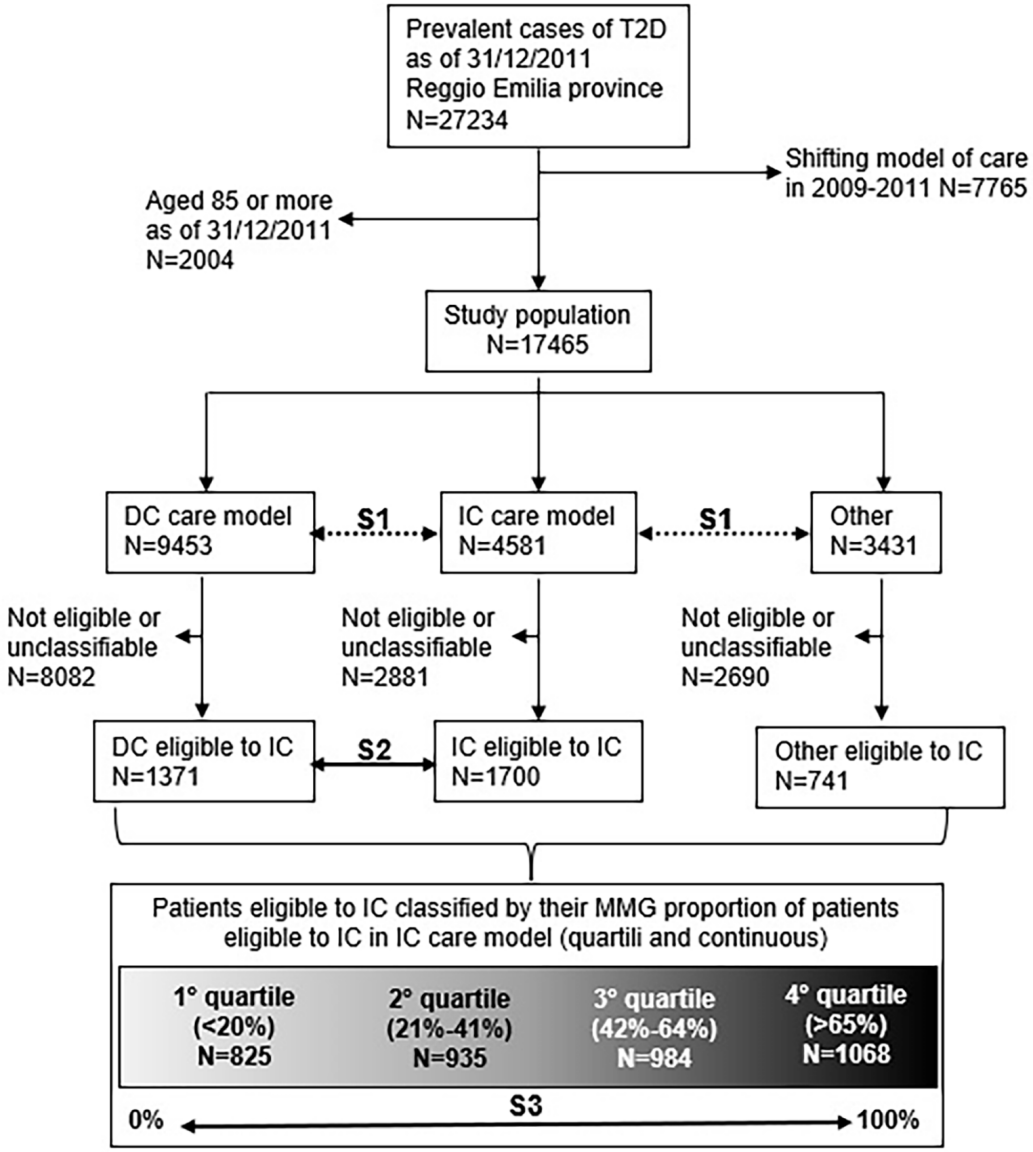
Flowchart showing the study population selection and the steps of analysis. S1 = step1 –mortality and hospitalisation rates by care model, no relative risk has been computed. S2 = step2 –to overcome selection bias, comparison was limited to patients eligible for the IC model, through mortality and hospitalisation risk rates, IC vs DC (Other-group vs DC analysis was omitted because the former was not a care model foreseen by the guideline). S3 = step3 –to overcome indication bias, we compared mortality and hospitalisation risk using the proportion of eligible patients actually in the IC model by GP (100% IC vs. 0% IC). DC = diabetes clinic; IC = integrated care.

On December 31, 2011, the REDR included 27,234 prevalent patients with T2D (5.3% out of the total resident population, Fig 1). Restricting the analysis only to T2D under the age of 85 and cared for by the same care model for at least 2 years, cases decreased to 17,465, of which 54.1% (N = 9453) in DC, 26.2% (N = 4581) in IC, and 19.7% (N = 3431) in the Other-group. Patients included in the IC model were older, with shorter time since diagnosis, better glucose control and better levels of other investigated serum tests than patients in the DC model (Table 1). The percentage of women was greater the IC model, and the percentage of foreigners was smaller. As expected, the percentage of insulin users among IC patients was very low. Looking at the characteristics of patients included in the Other-group, the most important result coming from the data was the low serum test rates compared to the other two groups, mainly from the HbA1c test, considered a primary test to check glucose control in T2D. BMI was not available for Other-group patients because the information was retrieved from the database of diabetes clinics. Distribution by care model and district of residence was very different, reflecting the different levels of implementation achieved by the IC model in 2009–11. Among the study population, one in four was unclassifiable with respect to IC eligibility criteria, there were 3,812 patients eligible for IC (21.8% of the total study population). Only slightly more than one third of the patients in IC were eligible for this care model, about one half were not eligible and the rest were unclassifiable. A median of 58 T2D patients were in the care of each GP and the interquartile range (IQR) was 47–70, while the median number of eligible patients was 12 and IQR 9–17, and the median number of unclassifiable patients was 13 and IQR 9–17.

**Table 1:** Baseline characteristics. Patient's baseline characteristics by care model. DC = diabetes clinic; IC = integrated care; variables were collected on 31/12/2011; only patients with T2D and aged <85 placed in the same care model for at least 2 years are included.

	All T2D (N = 17465)		DC (N = 9453; 54.1%)		IC (N = 4581; 26.2%)		Other (N = 3431; 19.7%)		P^
**Females N; %**	7656	43.8	4051	42.9	2032	44.4	1573	45.9	0,007
**Foreigners N; %**	866	5.0	552	5.8	108	2.4	206	6.0	<0.001
**Mean age; SD**	67.2	11.2	66.4	11.2	68.3	10.1	67.9	11.2	<0.001
**District of residence N; %**									<0.001
CMO	1338	7.7	807	8.5	345	7.5	186	5.4	
COR	1888	10.8	728	7.7	970	21.2	190	5.5	
GUA	2625	15.0	1280	13.5	999	21.8	346	10.1	
MON	2283	13.1	1371	14.5	454	9.9	458	13.4	
REG	6914	39.6	4330	45.8	1112	24.3	1472	42.9	
SCA	2417	13.8	937	9.9	701	15.3	779	22.7	
**Time since diagnosis mean; SD**	8.7	7.1	10.9	7.8	7.0	5.0	5.2	5.4	<0.001
HbA1c* **- %**									
No.—% of persons with data	13515	77.4	7580	80.2	4068	88.8	1867	45.6	<0.001
Mean % (mmol/mol); SD %	7.5 (59)	1.3	7.8 (62)	1.3	7.1 (54)	1.0	7.1 (54)	1.2	<0.001
BMI* **(kg/m2)**									
No.—% of persons with data	8994	51.5	6210	65.7	2784	60.8	0	0.0	<0.001
mean; SD	29.7	6.8	30.0	7.4	29.0	5.2	-	-	<0.001
**Triglycerides (mg/dl)**									
No.—% of persons with data	12054	69.0	6718	71.1	3560	77.7	1776	51.8	<0.001
mean; SD	138.6	84.6	143.5	90.5	129.0	70.8	139.0	85.3	<0.001
**LDL (mg/dl)**									
No.—% of persons with data	8667	49.6	4974	52.6	2609	57.0	1084	32.0	<0.001
mean; SD	100.1	32.6	97.9	32.6	99.9	31.0	110.4	34.8	<0.001
**GFR~ (ml/min/1.73m**^**2**^**)**									
No.—% of persons with data	12291	70.4	6802	72.0	3557	77.7	1932	56.3	<0.001
<60	2652	21.6	1726	25.4	572	16.1	354	18.3	<0.001
> = 60 N; %	9639	78.4	5076	74.6	2985	83.9	1578	81.7	
**Glucose-lowering medication N; %**									<0.001
None	4192	24.0	1365	14.4	1480	32.3	1347	39.3	
Oral drug	9209	52.7	4246	44.9	2965	64.7	1998	58.2	
Insulin	1949	11.2	1868	19.8	35	0.8	46	1.3	
Combined therapy	2115	12.1	1974	20.9	101	2.2	40	1.2	
**Eligible patients**^**#**^ **N; %**									<0.001
yes	3812	21.8	1371	14.5	1700	37.1	741	21.6	
no	9413	53.9	6279	66.4	2062	45.0	1072	31.2	
unclassifiable	4240	24.3	1803	19.1	819	17.9	1618	47.2	

^ p-value of the difference among care models

* last value in 2010–2011 period

~ When not available, we estimated glomerular filtration rate by using serum creatinine and Modification of Diet in Renal Disease (MDRD) Study equation.

# Eligible patients = those without previous hospitalisation for diabetes-related complications and without mild-severe renal complications and with HbA1c on target for IC setting and not rapid-acting insulin users.

When we compare the quartile with the lowest proportion of eligible patients actually included in IC and the highest quartile (Table 2), we go from <20% to >65% of eligible patients cared for in the IC model, and 78% of eligible patients actually included in IC are in the highest quartile. Distributions across quartiles of the proportion of IC-eligible patients actually included in IC by GPs was balanced in terms of age, time since diagnosis, HbA1c, BMI and triglycerides means, while the percentage of females decreased from the lowest to highest quartile (48.5% to 41.1%), as did the percentage of foreigners (3.1% to 2.3%). On the contrary, the number of available serum tests increased, as did the percentage of patients without glucose-lowering medication (the latter from 23.5% to 40.9%). Since activation of the IC model also depended on how the various health districts implemented organisational and logistical actions, there were significant differences in the distribution of quartiles between districts.

**Table 2:** Baseline characteristics by GPs’ use of integrated care model. GPs are classified according to the proportion of their IC-eligible patients who are included in the IC model. The eligible population is divided into quartiles according to this GP characteristic. The Table presents the covariates by quartiles of proportion of the GP's eligible patients included in IC model (1^st^ quartile lowest proportion of eligible patients included in IC model, 4^th^ quartile highest proportion).

	All eligible to IC (N = 3812)		1^st^ quartile (<20%)		2^nd^ quartile (21%-41%)		3^rd^ quartile (42%-64%)		4^th^ quartile >65%		P^
**N**	**3812**		**825**		**935**		**984**		**1068**		
**Distribution by care model N; %**											<0.001
DC	1371		504	61.1	401	42.9	316	32.1	150	14.0	
IC	1700		51	6.2	294	31.4	520	52.9	835	78.2	
Other	741		270	32.7	240	25.7	148	15.0	83	7.8	
**Females N; %**	1678	44.0	400	48.5	401	42.9	438	44.5	439	41.1	0.019
**Foreigners N; %**	85	2.2	26	3.1	24	2.6	10	1.0	25	2.3	0.045
**Mean Age; SD**	69.1	10.5	69.0	10.6	69.0	10.8	69.3	10.2	69.1	10.4	0.916
**District of residence N; %**											<0.001
CMO	411	10.8	90	10.9	52	5.6	117	10.6	172	15.3	
COR	443	11.6	0	0.0	16	1.7	41	5.2	400	35.5	
GUA	475	12.5	18	2.2	84	9.0	210	21.4	187	16.6	
MON	549	14.4	97	11.8	222	23.7	203	22.3	27	2.4	
REG	1436	37.7	500	60.6	409	43.7	356	35.2	170	15.1	
SCA	498	13.0	120	14.6	152	16.3	57	5.3	171	15.2	
**Time since diagnosis mean; SD**	7.1	5.9	7.3	6.6	7.2	6.6	7.1	5.4	6.8	5.2	0.313
HbA1c* **- %**											
No.—% of persons with data	3812	100.0	825	100.0	935	100.0	984	100.0	1068	100.0	
Mean (mmol/mol);SD	6.6 (49)	0.6	6.6 (49)	0.6	6.6 (49)	0.6	6.6 (49)	0.6	6.6 (49)	0.6	0.889
BMI* **(kg/m2)**											
No.—% of persons with data	1920	50.4	346	41.9	418	44.7	509	51.7	647	60.6	<0.001
mean; SD	29.0	6.3	29.3	9.3	28.9	5.2	28.7	5.5	29	5.6	0.663
**Triglycerides (mg/dl)**											
No.—% of persons with data	3567	69.0	755	91.5	878	93.9	927	94.2	1007	94.3	0.112
mean; SD	122.0	60.6	124.6	61.8	123.1	67.0	118.8	56.9	122.1	57.0	0.167
**LDL (mg/dl)**											
No.—% of persons with data	2570	67.4	491	59.5	643	68.8	684	69.5	752	70.4	0.083
mean; SD	100.6	31.1	101.9	30.0	104.1	32.4	99.9	30.7	100.7	31.1	0.013
**GFR~ (ml/min/1.73m**^**2**^**)**											
No.—% of persons with data	3812	100.0	825	100.0	935	100.0	984	100.0	1068	100.0	
<60											
> = 60 N; %	3812	100.0	825	100.0	935	100.0	984	100.0	1068	100.0	
**Glucose-lowering medication N; %**											<0.001
None	1282	33.6	194	23.5	297	31.8	354	36.0	437	40.9	
Oral drug	2380	62.4	593	71.9	607	64.9	589	59.9	591	55.3	
Insulin	37	1.0	7	0.9	11	1.2	12	1.2	7	0.7	
Combined therapy	113	3.0	31	3.8	20	2.1	29	2.9	33	3.1	

^ p-value of the difference among care models

* last value in 2010–2011 period

~ when not available, we estimated glomerular filtration rate by using serum creatinine and Modification of Diet in Renal Disease (MDRD) Study equation.

All-cause mortality standardised rates for patients in the DC care model were double those in IC (Table 3); the all-cause mortality standardised rate for those in the Other-group was between the other two (DC: 44.0, 95%CI 42.0–45.9; IC: 24.5, 95%CI 22.5–26.5; Other-group: 30.4, 95%CI 27.8–33.0, per 1000 person-year). In the comparison between the DC and IC models, the pattern was similar for all investigated causes except for AMI, where the gap increased slightly (DC: 2.2, 95%CI 1.8–2.7; IC: 0.9, 95%CI 0.5–1.3; per 1000 person-year), and for cerebrovascular diseases, where the gap decreased slightly (DC: 3.6, 95%CI 3.0–4.1; IC: 2.3, 95%CI 1.7–3.0; per 1000 person-year). The comparison between patients in the DC care model and Other-group was more erratic, with the lowest gap observed for cerebrovascular diseases (DC: 3.6, 95%CI 3.0–4.1; Other-group: 2.6, 95%CI 1.9–3.4; per 1000 person-year). Standardised rates for all-case hospitalisations in DC were double those of the IC model (Table 3), and the rate for the Other-group was between the other two (DC: 437.3, 95%CI 432.3–442.2; IC: 252.0, 95%CI 245.9–258.1; Other-group: 294.4, 95%CI 287.0–301.9, per 1000 person-year). The difference was particularly strong for hypoglycemic coma (DC: 0.3, 95%CI 0.1–0.5; IC: 0.05, 95%CI 0.00–0.14; per 1000 person-year) and acute hyperglycaemic complications (DC: 0.7, 95%CI 0.4–0.9; IC: 0.09, 95%CI 0.00–0.22; per 1000 person-year).

**Table 3:** Mortality and hospitalization rates. Number of events by care model–crude and standardised rates, with 95% confidence interval (95%CI). DC = diabetes clinic; IC = integrated care.

**MORTALITY**	**DC**					**IC**					**Other**				
	**N**	**PY**	**crude rate (x10^3^)**	**Std rate” (x10^3^)**	**95%CI**	**N**	**PY**	**crude rate (x10^3^)**	**std rate” (x10^3^)**	**95%CI**	**N**	**PY**	**crude rate (x10^3^)**	**std rate” (x10^3^)**	**95%CI**
all causes	1771	42204	42.0	44.0	(42.0–45.9)	553	21451	25.8	24.5	(22.5–26.5)	512	15731	32.6	30.4	(27.8–33.0)
cardiovascular	562		13.3	14.0	(12.9–15.2)	161		7.5	7.2	(6.1–8.3)	172		10.9	10.0	(8.5–11.5)
AMI	90		2.1	2.2	(1.8–2.7)	19		0.9	0.9	(0.5–1.3)	23		1.5	1.4	(0.8–2.0)
cerebrovascular	143		3.4	3.6	(3.0–4.1)	53		2.5	2.3	(1.7–3.0)	56		2.9	2.6	(1.9–3.4)
diabetes	198		4.7	4.9	(4.2–5.6)	54		2.5	2.4	(1.8–3.1)	47		3.0	2.8	(2.0–3.6)
Renal	46		1.1	1.1	(0.8–1.5)	13		0.6	0.6	(0.3–0.9)	10		0.6	0.6	(0.2–1.0)
HOSPITALISATION*	**N**	**PY**	**crude rate (x10**^**3**^**)**	**std rate” (x10**^**3**^**)**	**95%CI**	**N**	**PY**	**crude rate (x10**^**3**^**)**	**std rate” (x10**^**3**^**)**	**95%CI**	**N**	**PY**	**crude rate (x10**^**3**^**)**	**std rate” (x10**^**3**^**)**	**95%CI**
all causes	14946	35074	426.1	437.3	(432.3–442.2)	4729	18073	261.7	252.0	(245.9–258.1)	4019	13319	301.7	294.4	(287.0–301.9)
cardiovascular	4513	38618	116.9	121.3	(118.0–124.4)	1233	20167	61.1	58.1	(55.0–61.3)	1149	14649	78.4	75.9	(71.7–80.1)
AMI	577	41526	14.0	14.2	(13.1–15.4)	159	21238	7.5	7.3	(6.1–8.4)	157	15506	10.1	10.0	(8.5–11.7)
cerebrovascular	938	40935	22.9	23.9	(22.4–25.4)	274	21048	13.0	12.3	(10.9–13.7)	254	15357	16.5	15.9	(14.0–17.9)
hypoglycaemic coma	12	42185	0.2	0.3	(0.1–0.5)	1	21451	0.1	0.05	(0.00–0.14)	1	15730	0.1	0.1	(0.00–0.17)
acute hyperglycaemic complications	27	42156	0.6	0.7	(0.4–0.9)	2	21451	0.1	0.09	(0.00–0.22)	5	15721	0.3	0.3	(0.03–0.6)
Lower limbs amputations	131	42038	3.1	3.2	(2.6–3.7)	19	21437	0.9	0.8	(0.4–0.12)	31	15700	2.0	1.9	(1.3–2.6)
renal complications	528	41807	12.6	12.7	(11.6–13.8)	80	21361	3.7	3.7	(2.9–4.5)	85	15669	5.4	5.3	(4.1–6.4)
ocular complications	91	42033	2.7	2.1	(1.7–2.6)	16	21414	0.7	0.7	(0.4–1.0)	14	15712	0.9	0.9	(0.4–1.3)

" standardized by sex and age using all T2D population person-years

* excluding day hospital and keeping principal diagnosis

Restricting the analysis to IC-eligible patients actually placed in the DC or IC model (Table 4), all-cause mortality rate ratios still showed a reduced risk of 40% for IC patients compared to the DC model (adjusted IRR: 0.62, 95%CI 0.51–0.76). The same, even more pronounced, pattern was observed for all the investigated groups of causes. In the case of all-cause hospital admissions, the risk was almost 25% lower for IC patients than for DC patients (adjusted IRR: 0.74, 95%CI 0.66–0.82), and the gap was particularly wide for renal complications (adjusted IRR: 0.31, 95%CI 0.15–0.65).

**Table 4:** Mortality and hospitalisation rate ratios by care model. Mortality and hospitalisation rate ratios, with 95% confidence interval (95%CI), for all and diabetes-related causes for patients in DC and IC model. Analyses restricted to IC-eligible patients. DC = diabetes clinic; IC = integrated care; DC was used as reference.

**MORTALITY**	**DC (N = 1371)**		**IC (N = 1700)**		**unadjusted MRR (95%CI)**	**adjusted MRR (95%CI)”**	***Other (N = 741)***	
	**N**	**PY**	**N**	**PY**			***N***	***PY***
**all causes**	231	6302	184	8083	0.62 (0.50–0.76)	0.62 (0.51–0.76)	*112*	*3434*
cardiovascular	75		47		0.49 (0.33–0.71)	0.51 (0.35–0.74)	*38*	
AMI	11		6		0.43 (0.16–1.15)	0.22 (0.18–1.40)	*3*	
cerebrovascular	23		12		0.41 (0.20–0.82)	0.43 (0.21–0.88)	*8*	
diabetes	22		12		0.43 (0.21–0.88)	0.43 (0.20–0.92)	*7*	
renal	2		5		-	-	*2*	
HOSPITAL ADMISSIONS*	**CD**		**IC**		**unadjusted IRR (95%CI)**	**adjusted IRR (95%CI)”**	***Other***	
	**N**	**PY**	**N**	**PY**			***N***	***PY***
**all causes**	741	4515	777	6229	0.76 (0.68–0.85)	0.74 (0.66–0.82)	*361*	*2584*
cardiovascular	286	5647	257	7526	0.67 (0.56–0.81)	0.66 (0.56–0.79)	*121*	*3191*
AMI	35	6240	32	8023	0.71 (0.44–1.15)	0.80 (0.48–1.31)	*17*	*3394*
cerebrovascular	117	6054	83	7904	0.54 (0.41–0.72)	0.54 (0.40–0.72)	*38*	*3359*
hypoglycaemic coma	0	6302	1	8082	-	-	*1*	*3434*
acute hyperglycaemic complications	1	6298	2	8082	-	-	*2*	*3433*
lower limbs amputations	7	6286	3	8074	-	-	*1*	*3434*
renal complications	26	6264	10	8067	0.30 (0.14–0.62)	0.31 (0.15–0.65)	*12*	*3424*
ocular complications	2	6295	4	8071	-	-	*2*	*3425*

" adjusting covariates were sex, citizenship, age, years since diagnosis (the last two as continuous variables)

* first hospitalisation during follow up, excluding day hospital and keeping principal diagnosis

When we adopted the instrumental variable approach using the proportion of IC-eligible patients actually included in the IC model by GPs as a main predictor (Table 5), the estimated rate ratios for all-cause mortality was 0.83 (95%CI 0.60–1.13) and 0.95 (95%CI 0.54–1.70) for cardiovascular diseases, under the hypothesis of a linear association between the outcomes and the instrumental variable and comparing a GP with 0% of eligible patients in IC vs. a GP with 100%. For diabetes mortality, there was still a decreased risk, largely compatible with random fluctuations. Similarly, the rate ratios for all-cause hospitalisations of a GP with 0% of eligible patients in IC vs. a GP with 100% were 0.90 (95%CI 0.76–1.06) and 0.91 (95%CI 0.69–1.20), respectively, for cardiovascular diseases. Regarding renal complications, the risk was reduced for GPs with high proportion of patients in IC, but the difference was compatible with random fluctuations.

**Table 5:** Mortality and hospitalisation rate ratios by GPs’ use of integrated care model. Mortality and hospitalisation rate ratios, with 95% confidence interval (95%CI), for all and diabetes-related causes, comparing the proportion of each GP's eligible patients included in IC model. Analyses restricted to patients eligible for IC (N = 3812).

**MORTALITY**	**PY**	**N**	**estimated MRR and IRR for 100% IC vs. 0% IC among eligible**	
			**unadjusted MRR (95%CI)^**	**adjusted MRR (95%CI)”**
**all causes**	17819	527	0.80 (0.58–1.11)	0.83 (0.60–1.13)
cardiovascular		160	0.89 (0.52–1.56)	0.95 (0.54–1.70)
AMI		20	0.84 (0.17–4.12)	0.97 (0.19–4.91)
cerebrovascular		43	1.05 (0.35–3.10)	1.15 (0.38–3.50)
Diabetes		41	0.32 (0.10–0.99)	0.34 (0.11–1.08)
HOSPITAL ADMISSIONS*			**unadjusted IRR (95%CI)^**	**Adjusted IRR (95%CI)”**
**all causes**	13328	1879	0.90 (0.76–1.06)	0.90 (0.76–1.06)
cardiovascular	16364	664	0.90 (0.69–1.19)	0.91 (0.69–1.20)
AMI	17657	84	0.78 (0.36–1.69)	0.83 (0.38–1.83)
cerebrovascular	17317	238	0.85 (0.53–1.34)	0.87 (0.55–1.39)
renal complications	17755	48	0.40 (0.14–1.13)	0.41 (0.11–1.16)

^ Poisson model using IC proportion among eligibles by GP (continuous variable) as explanatory variable

" adjusting covariates were sex, citizenship, age, years since diagnosis (the last two as continuous variables)

* first hospitalisation during follow up, excluding day hospital and keeping principal diagnosis

## Discussion

Our population-based cohort study using an instrumental variable approach showed that the IC model probably is not inferior to the DC care model for the main investigated clinical outcomes. It showed negligible effects on mortality and hospital admissions. The progressive steps of the analysis (i.e., from current patients’ distribution by care model to instrumental variable approach) allowed us to overcome both the selection and indication bias. Results from models adjusted by the main clinical characteristics, and also restricting the population to only those eligible, still showed a large difference in mortality and hospitalisation in favour of the IC model, but when an analysis similar to randomisation was adopted the differences almost disappeared.

How to manage chronic patients is one of the main issues of this century. Many health systems in Europe and in the US have begun to involve primary care services more actively to reduce the workload of specialist clinics, to reduce patients’ physical and personal distance to increase access to care, and finally, to favour proactive medicine interventions to prevent diabetes complications. The IC model implemented in Reggio Emilia was inspired by these principles. Nevertheless, the choice of a non-specialist setting for a disease as complex as diabetes could be not justified even in the presence of all these possible advantages if it resulted in less safe and less effective care.

The relevance of the question is confirmed by a recent Cochrane systematic review, conducted to determine the effectiveness of shared care health service interventions designed to improve the management of chronic disease across the primary/specialty care interface [24]. Our results are consistent with the conclusions of this review. Specifically, among the main results, the systematic review highlights that the studies probably showed little or no difference in hospital admissions (with evidence of moderate certainty). As far as diabetes is concerned, one included RCT [10] found no significant differences in unscheduled admissions, one [25] found no significant differences in the number of patients admitted to hospital for a diabetes-related reason, and number of deaths. A third RCT [26] included in the review was not taken into consideration because it compared all-cause mortality between “specialty care” (i.e., consultation care provided by an endocrinologist or a general internist in concert with the patient’s primary care doctor) and “primary care” (cared for by family doctors alone). A further RCT aimed at evaluating the quality of a shared care programme for patients with T2D; providing evidence on the advantages and disadvantages compared with a programme in a specialised outpatient clinic is ongoing and the results are still unpublished [27].

Our starting hypothesis is that IC is not inferior to the DC care model in controlling diabetes-related morbidity and mortality, even though it should consume fewer resources and be less time consuming for the patient.

Indeed, an analysis of the current patients’ distribution by care model, mortality and hospital admission showed that rates were higher for patients in DC care model compared to their IC counterparts. The guidelines recommend caring for low-risk profile T2D patients in the IC model. Therefore, by definition, patients included in the IC are those at lower risk of complications, hospitalisation and of death than the other T2D patients [6]. Although different factors such as physician and patient preferences and medical or social patient frailty can influence the decision to care for T2D patients in the IC model, this was an expected result and is indirect evidence of adherence to guidelines.

Even patients included in the Other-group showed lower rates compared to their DC counterparts. This latter group is heterogeneous, and includes neglected patients belonging to difficult-to-reach populations, subjects being treated in other structured care models not specifically dedicated to diabetes (patients institutionalised or cared for at home for other chronic diseases), subjects who have opted out of any structured model because they prefer to be treated only by their GP or because they have a private diabetologist, and finally, newly diagnosed patients who have yet to choose a care model.

The results from previous observational studies on this topic are consistent with our analyses based on real-world data. Compared to the most similar studies [28,29], we found a lower proportion of patients who are not in a DC or IC model and higher mortality rates for each model of care. Possibly, the introduction of the DC and IC model in our province since 2006 has enhanced the accuracy in assigning patients to one of the two care models. Differences in standardised mortality rates can be due to the population used for standardising (the general population for the Turin study, while we used the population with diabetes) and inclusion criteria of the study population (our study is registry-based, while the other two studies defined a cohort based on administrative data). Both previous studies focused on comparing patients under structural care (IC and DC) and patients cared for by GPs alone, and showed higher mortality [28,29] and hospitalisation [29] for the latter group. Nevertheless, authors of the Baldo study [28] found lower mortality risks for IC compared to DC.

In our study, we chose to restrict the analysis to eligible patients to minimise selection bias induced by the presence of strict criteria to define IC-eligible patients. If the guidelines were applied in a uniform and complete manner and no other factors influenced the choice of assigning a patient to one care model rather than another, one would expect to find no eligible patients in the DC care model and all patients in the IC model defined as eligible. In fact, 21.8% of the subjects in our study population were IC-eligible, with the percentage varying across care models: 14.5% for DC, 37.1% for IC, and 21.6% for GP. Even when we restrict the comparison to IC-eligible patients, those actually cared for in IC experienced lower mortality and hospitalization risks than those cared for in the DC; for mortality, the reduction was consistent for all-causes, cardiovascular, cerebrovascular and diabetes, and for hospitalisation, for all-causes, cardiovascular, cerebrovascular, and renal complications. The difference is plausibly due to unregistered prognostic factors that were at the basis of the physician’s decision to include or not include the patient in a low-risk model of care.

Finally, we adopted an instrumental variable analysis approach to minimise indication bias, i.e., to take into account latent factors such as physician and patient preferences and opportunity, as well as unregistered prognostic factors. We used the proportion of IC-eligible patients that were actually included by GPs in the IC model as an instrumental variable. We assumed that the case mix for each GP would be an almost random sample of the population. Indeed, we showed that mean age, time since diagnosis, HbA1c, mean BMI and triglycerides were balanced among each GP’s eligible patients, which supports the validity of our instrumental variable. Only the availability of a serum test was not balanced, but this variable is clearly linked to the care model and to the GP’s attitudes (i.e., ability to keep up to date, initiative, aptitude for teamwork), thus it is not surprising that it varies between GPs. Fortunately, not all the variability between GPs is due to each GP’s characteristics: the activation of the IC model differed according to other organisational, logistical and timing factors (i.e., information technology facilities, contracts with GPs, differences in protocols of T2D management among diabetes clinics), as is clear if we look at the differences in proportion to IC-eligible patients actually included in the IC model between districts.

### Strengths and limitation

The main limitation of this method is that we are using an ecological variable instead of an individual one, thus losing statistical power (the 95%CI gets larger in Table 5). We are also misclassifying patients, since our instrumental variable is a continuous variable; except for those GPs who have 0% or 100% of eligible patients included in the IC model, we are considering IC-eligible patients that are actually cared for by one definite care model as cared for by % of IC. The loss of power is quantified by the precision of our estimates and we can say that it is acceptable for all-cause hospitalisation and for all-cause mortality. For specific cause of death and hospitalisation, the estimates are very imprecise. The loss of discriminating power due to the use of an ecological variable depends on the variance in the proportion of patients in the IC models between GPs; the lowest quartile has less than 20% of IC-eligible patients in the IC model and the highest quartile more than 65% of IC-eligible patients are in the IC model.

Moreover, although our study included almost everyone with T2D living in the Reggio Emilia province, it is still underpowered to perform a formal non-inferiority test, which would assess acceptable differences in the considered major outcomes.

This cohort study is population based and includes the results of implementing the IC model as it took place in all the resident population of IC-eligible patients in the province of Reggio Emilia. Furthermore, for our analysis, we considered only patients with T2D treated by the same care model for at least two years before the start of follow up, excluding those who shifted from one model to another. This choice was necessary because by definition, any worsening of the disease could imply a shift from IC to DC. Thus, most of the study outcomes occurred when the patient was in the DC model, even if resulting from the care received during the period when the patient was in the IC model. On the other hand, this choice introduced further misclassification of the exposure, as in any intention-to-treat analysis, driving our results toward a null hypothesis.

## Conclusions

In low-risk patients with T2D, the integrated care model involving both GPs and diabetes clinic professionals showed similar mortality and hospitalisation risks as a model with higher use of specialized care, with exclusive patient management by the diabetes clinic. This was a necessary, but not sufficient, condition to prove the effectiveness of the integrated care model. Further research is needed to assess whether this model, aimed at reducing the workload of specialist care, increasing accessibility and facilitating proactive and initiative medicine through the involvement of primary care physicians, is actually less resource-consuming and more acceptable for patients.

The comparison between two care models with observational studies is affected by strong biases that systematically favour the one that should be prescribed to less complicated and less severe cases.

## Supporting information

S1 Fig Study timeline chart. *HbA1c and FGR last value were both integrated care eligibility criteria and baseline characteristics.
(TIF)

S1 Table Diabetes-related diagnosis ICD-IX codes. (PDF)

## References

[1] Guariguata L, Whiting DR, Hambleton I, Beagley J, Linnenkamp U, Shaw JE. Global estimates of diabetes prevalence for 2013 and projections for 2035. Diabetes Res Clin Pract [Internet]. 2014;103(2) doi: 10.1016/j.diabres.2013.11.002:137–49 24630390 10.1016/j.diabres.2013.11.002

[2] Ballotari P, Chiatamone Ranieri S, Vicentini M, Caroli S, Gardini A, Rodolfi R, et al Building a population-based diabetes register: An Italian experience. Diabetes Res Clin Pract [Internet]. 2014;103(1) doi: 10.1016/j.diabres.2013.11.020:79–87 24369984 10.1016/j.diabres.2013.11.020

[3] ISTAT. Diabetes in Italy. Years 2000–2016. [Internet]. 2017 [cited 2017 Oct 9]. Available from: https://www.istat.it/en/files/2017/07/Report_Diabetes_En_def.pdf?title=Diabetes+in+Italy+-+24+Jul+2017+-+Full+text.pdf

[4] Global Burden of Disease Study 2013 Collaborators. Global, regional, and national incidence, prevalence, and years lived with disability for 301 acute and chronic diseases and injuries in 188 countries, 1990–2013: a systematic analysis for the Global Burden of Disease Study 2013. Lancet [Internet]. 2015;386(9995) doi: 10.1016/S0140-6736(15)60692-4:743–800 10.1016/S0140-6736(15)60692-4 PMC4561509 26063472

[5] Fox CS, Golden SH, Anderson C, Bray GA, Burke LE, de Boer IH, et al Update on Prevention of Cardiovascular Disease in Adults With Type 2 Diabetes Mellitus in Light of Recent Evidence: A Scientific Statement From the American Heart Association and the American Diabetes Association. Diabetes Care [Internet]. 2015;38(9) doi: 10.2337/dci15-0012:1777–803 26246459 10.2337/dci15-0012 PMC4876675

[6] Emilia-Romagna Regional Commette. Linee guida regionali per la gestione integrata del diabete mellito tipo 2. [Internet]. 2009. Available from: http://salute.regione.emilia-romagna.it/documentazione/leggi/regionali/linee-guida/linee-guida-regionali-per-la-gestione-integrata-del-diabete-mellito-tipo-2-aggiornamento-dellimplementazione/view

[7] World Health Organization. Innovative care for chronic conditions: building blocks for action: global report / [Health Care for Chronic Conditions Team] [Internet]. Geneva; 2002 [cited 2017 Oct 9] doi: ISBN 9241590173. Available from: http://www.who.int/chp/knowledge/publications/icccglobalreport.pdf?ua=1

[8] Smith SM, Cousins G, Clyne B, Allwright S, O’Dowd T. Shared care across the interface between primary and specialty care in management of long term conditions In: Smith SM, editor. Cochrane Database of Systematic Reviews [Internet]. Chichester, UK: John Wiley & Sons, Ltd; 2017 doi: 10.1002/14651858.CD004910.pub3 p. CD004910. 10.1002/14651858.CD004910.pub3 PMC6473196 28230899

[9] Smith SA, Shah ND, Bryant SC, Christianson TJH, Bjornsen SS, Giesler PD, et al Chronic Care Model and Shared Care in Diabetes: Randomized Trial of an Electronic Decision Support System. Mayo Clin Proc [Internet]. 2008;83(7) doi: 10.4065/83.7.747:747–57 18613991 10.4065/83.7.747

[10] DICE Team. Integrated care for diabetes: clinical, psychosocial, and economic evaluation. Diabetes Integrated Care Evaluation Team. BMJ [Internet]. 1994;308(6938) doi: 10.1136/bmj.308.6938.1208:1208–12 PMC2540045 8180540

[11] Duran A, Runkle I, Matía P, de Miguel MP, Garrido S, Cervera E, et al Family physician and endocrinologist coordination as the basis for diabetes care in clinical practice. BMC Endocr Disord [Internet]. 2008;8 doi: 10.1186/1472-6823-8-9:9 18671870 10.1186/1472-6823-8-9 PMC2518542

[12] Goderis G, Borgermans L, Grol R, Van Den Broeke C, Boland B, Verbeke G, et al Start improving the quality of care for people with type 2 diabetes through a general practice support program: A cluster randomized trial. Diabetes Res Clin Pract [Internet]. 2010;88(1) doi: 10.1016/j.diabres.2009.12.012:56–64 20047770 10.1016/j.diabres.2009.12.012

[13] Hoskins PL, Fowler PM, Constantino M, Forrest J, Yue DK, Turtle JR. Sharing the care of diabetic patients between hospital and general practitioners: does it work? Diabet Med [Internet]. 10(1) doi: 10.1111/j.1464-5491.1993.tb02001.x:81–6 10.1111/j.1464-5491.1993.tb02001.x 8435994

[14] Katon WJ, Lin EHB, Von Korff M, Ciechanowski P, Ludman EJ, Young B, et al Collaborative Care for Patients with Depression and Chronic Illnesses. N Engl J Med [Internet]. 2010;363(27) doi: 10.1056/NEJMoa1003955:2611–20 21190455 10.1056/NEJMoa1003955 PMC3312811

[15] Katon WJ, Von Korff M, Lin EHB, Simon G, Ludman E, Russo J, et al The Pathways Study. Arch Gen Psychiatry [Internet]. 2004;61(10) doi: 10.1001/archpsyc.61.10.1042:1042 15466678 10.1001/archpsyc.61.10.1042

[16] Scherpbier-de Haan ND, Vervoort GM, van Weel C, Braspenning JC, Mulder J, Wetzels JF, et al Effect of shared care on blood pressure in patients with chronic kidney disease: a cluster randomised controlled trial. Br J Gen Pract [Internet]. 2013;63(617) doi: 10.3399/bjgp13X675386:798–806 10.3399/bjgp13X675386 PMC3839388 24351495

[17] Smith S, Bury G, O’Leary M, Shannon W, Tynan A, Staines A, et al The North Dublin randomized controlled trial of structured diabetes shared care. Fam Pract [Internet]. 2004;21(1) doi: 10.1093/fampra/cmh109:39–45 10.1093/fampra/cmh109 14760042

[18] Schneeweiss S, Seeger JD, Jackson JW, Smith SR. Methods for comparative effectiveness research/patient-centered outcomes research: from efficacy to effectiveness. J Clin Epidemiol [Internet]. 2013;66 (8 Suppl) doi: 10.1016/j.jclinepi.2013.05.012:S1-4 23849143 10.1016/j.jclinepi.2013.05.012

[19] Prentice RL, Langer R, Stefanick ML, Howard B V, Pettinger M, Anderson G, et al Combined Postmenopausal Hormone Therapy and Cardiovascular Disease: Toward Resolving the Discrepancy between Observational Studies and the Women’s Health Initiative Clinical Trial. Am J Epidemiol [Internet]. 2005;162(5) doi: 10.1093/aje/kwi223:404–14 16033876 10.1093/aje/kwi223

[20] Petitti DB, Freedman DA. Invited Commentary: How Far Can Epidemiologists Get with Statistical Adjustment? Am J Epidemiol [Internet]. 2005;162(5) doi: 10.1093/aje/kwi224:415–8 16014781 10.1093/aje/kwi224

[21] Stukel TA, Fisher ES, Wennberg DE, Alter DA, Gottlieb DJ, Vermeulen MJ. Analysis of Observational Studies in the Presence of Treatment Selection Bias. JAMA [Internet]. 2007;297(3) doi: 10.1001/jama.297.3.278:278 17227979 10.1001/jama.297.3.278 PMC2170524

[22] Guo Z, Cheng J, Lorch SA, Small DS. Using an instrumental variable to test for unmeasured confounding. Stat Med [Internet]. 2014;33(20) doi: 10.1002/sim.6227:3528–46 24930696 10.1002/sim.6227 PMC4145076

[23] Levey AS, Coresh J, Greene T, Stevens LA, Zhang YL, Hendriksen S, et al Using standardized serum creatinine values in the modification of diet in renal disease study equation for estimating glomerular filtration rate. Ann Intern Med [Internet]. 2006;145(4) doi: 10.7326/0003-4819-145-4-200608150-00004:247–54 10.7326/0003-4819-145-4-200608150-00004 16908915

[24] Smith SM, Cousins G, Clyne B, Allwright S, O’Dowd T. Shared care across the interface between primary and specialty care in management of long term conditions In: Smith SM, editor. Cochrane Database of Systematic Reviews [Internet]. Chichester, UK: John Wiley & Sons, Ltd; 2017 doi: 10.1002/14651858.CD004910.pub3 p. CD004910. 10.1002/14651858.CD004910.pub3 PMC6473196 28230899

[25] Hurwitz B, Goodman C, Yudkin J. Prompting the clinical care of non-insulin dependent (type II) diabetic patients in an inner city area: one model of community care. BMJ [Internet]. 1993;306(6878):624–30. 8461815 10.1136/bmj.306.6878.624 PMC1676949

[26] McAlister FA, Majumdar SR, Eurich DT, Johnson JA. The effect of specialist care within the first year on subsequent outcomes in 24,232 adults with new-onset diabetes mellitus: population-based cohort study. Qual Saf Health Care [Internet]. 2007;16(1) doi: 10.1136/qshc.2006.018648:6–11 17301194 10.1136/qshc.2006.018648 PMC2464930

[27] Munch L, Bennich B, Arreskov AB, Overgaard D, Konradsen H, Knop FK, et al Shared care management of patients with type 2 diabetes across the primary and secondary healthcare sectors: study protocol for a randomised controlled trial. Trials [Internet]. 2016;17(1) doi: 10.1186/s13063-016-1409-y:277 27259669 10.1186/s13063-016-1409-y PMC4893266

[28] Baldo V, Lombardi S, Cocchio S, Rancan S, Buja A, Cozza S, et al Diabetes outcomes within integrated healthcare management programs. Prim Care Diabetes [Internet]. 2015;9(1) doi: 10.1016/j.pcd.2014.03.005:54–9 24746417 10.1016/j.pcd.2014.03.005

[29] Giorda C, Picariello R, Nada E, Tartaglino B, Marafetti L, Costa G, et al The impact of adherence to screening guidelines and of diabetes clinics referral on morbidity and mortality in diabetes. Atkin SL, editor. PLoS One [Internet]. 2012;7(4) doi: 10.1371/journal.pone.0033839:e33839 22509263 10.1371/journal.pone.0033839 PMC3317933

