# Supplementary figures and images for: Effectiveness of integrated care model for type 2 diabetes: A population-based study in Reggio Emilia (Italy)
Source: PLoS One. 2018 Mar 27;13(3):e0194784. doi: 10.1371/journal.pone.0194784 (PMC5870991; doi:10.1371/journal.pone.0194784)

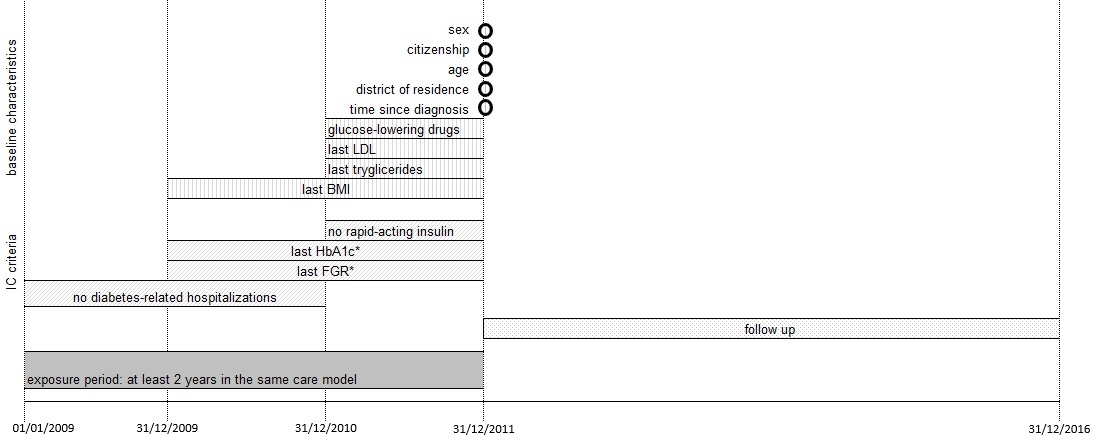

Supplement: S1 Fig — *HbA1c and FGR last value were both integrated care eligibility criteria and baseline characteristics. (TIF) [file pone.0194784.s001.tif]
